# Soil Fungal Community Composition, Not Assembly Process, Was Altered by Nitrogen Addition and Precipitation Changes at an Alpine Steppe

**DOI:** 10.3389/fmicb.2020.579072

**Published:** 2020-10-16

**Authors:** Yuanming Xiao, Changbin Li, Yang Yang, Yunfeng Peng, Yuanhe Yang, Guoying Zhou

**Affiliations:** ^1^Northwest Institute of Plateau Biology, Chinese Academy of Sciences, Xining, China; ^2^College of Life Sciences, University of Chinese Academy of Sciences, Beijing, China; ^3^State Key Laboratory of Vegetation and Environmental Change, Institute of Botany, Chinese Academy of Sciences, Beijing, China; ^4^Key Laboratory of Tibetan Medicine Research, Chinese Academy of Sciences, Xining, China; ^5^Qinghai Key Laboratory of Qinghai-Tibetan Plateau Biological Resources, Xining, China

**Keywords:** soil fungi, phylogenetic community structure, community assembly, climate change, structural equation model

## Abstract

Global climate change and nitrogen deposition have been having broad impacts on microorganisms. On the Qinghai-Tibetan Plateau (QTP), the responses of soil microbial community assemblage and diversity to nitrogen deposition and changes in precipitation are poorly understood, especially in the alpine steppe. In this study, we conducted a field manipulative experiment of nitrogen deposition and precipitation amount in an alpine steppe on the northeastern QTP and investigated the responses of community composition, diversity, and community assemblage of soil fungi. Soil fungal community compositions were significantly altered under nitrogen addition, precipitation change, and their interaction, and positively related with soil moisture, soil pH, and plant species richness. However, they were negatively related to soil mineralizable N and soil available P content. Operational taxonomic units (OTU) richness and Chao 1 index decreased under nitrogen addition combined with precipitation reduction treatment, whereas the Shannon–Wiener index declined only under precipitation increment treatment. Convergent fungal community assembly processes were not acutely altered by both nitrogen addition and precipitation changes, indicating that environmental filtering was a dominant ecological process controlling fungal community assemblage. By elucidating the above questions, the study enhanced our ability to predict the responses of soil fungal communities to nitrogen deposition and precipitation changes at alpine steppes on the QTP in the future.

## Introduction

Dominant ecological processes controlling soil microbial community assemblage have attracted extensive attention (Dini-Andreote et al., 2015). Evaluating the relative importance of deterministic and stochastic ecological processes in community assembly is a primary work for exploring community assembly (Dumbrell et al., 2010; Nemergut et al., 2013; Mori et al., 2015; Feng et al., 2018; Luo et al., 2019; Gao et al., 2020). For example, Beck et al. (2015) found that the deterministic and stochastic ecological processes jointly controlled fungal community assembly. Global changes have broad and profound impacts on microbial communities (Hutchins et al., 2019); for example, Chen et al. (2017) revealed that nitrogen addition enhanced the role of deterministic processes (i.e., environmental filtering) during soil arbuscular mycorrhizal fungal community assemblage in a temperate steppe. The Qinghai-Tibetan Plateau (QTP) is the largest and highest plateau in the world (average elevation, >4,000 m; area, 2.5 × 10^6^ km^2^), and low temperature and strong evaporation are primary climatic features (Zheng and Zhao, 2017), which limit the rate of mineralization of soil organic matter and make this area more sensitive to global changes (e.g., nitrogen deposition and precipitation change). Soil fungi play important roles in accelerating biogeochemical cycles (Amend et al., 2015), maintaining the health of soil ecosystems (Frac et al., 2018), and regulating feedback between plants and soil (Semchenko et al., 2018) and are studied well in a global scale (Tedersoo et al., 2014; Davison et al., 2015), whereas it is still poorly understood on the QTP. Therefore, it is important that the responses of soil fungal diversity, community composition, and assemblage process to nitrogen deposition and precipitation change are explored on the QTP. Human activity-induced nitrogen deposition has triggered a lot of changes in ecological processes and environmental factors, such as soil available nitrogen, soil pH, availability of soil phosphorus, and negative effects on biodiversity (Serna-Chavez et al., 2013; Lu et al., 2014; Chen et al., 2017). Soil environmental variables strongly affected soil fungal communities (Shen et al., 2013; Wang et al., 2017; Cho et al., 2019). On the QTP, nitrogen deposition has been presenting an increasing trend gradually, ranging from 0.87 to 1.38 g N m^–2^ year^–1^ in the recent decade (Lü and Tian, 2007). Thus, soil fungi diversity might decline, and community composition might change significantly in the future. However, we still poorly understand how soil fungal diversity, community composition, and assemblage process respond to nitrogen deposition, and relationships between fungal community composition and soil environmental variables remain to be unclear. In addition to soil variables, plant diversity was also a factor affecting soil fungal community (Chen et al., 2017). Thus, examining relationships between soil fungal community and plant diversity is also important for comprehensively understanding the responses of soil fungal community to nitrogen deposition.

**FIGURE 1:**
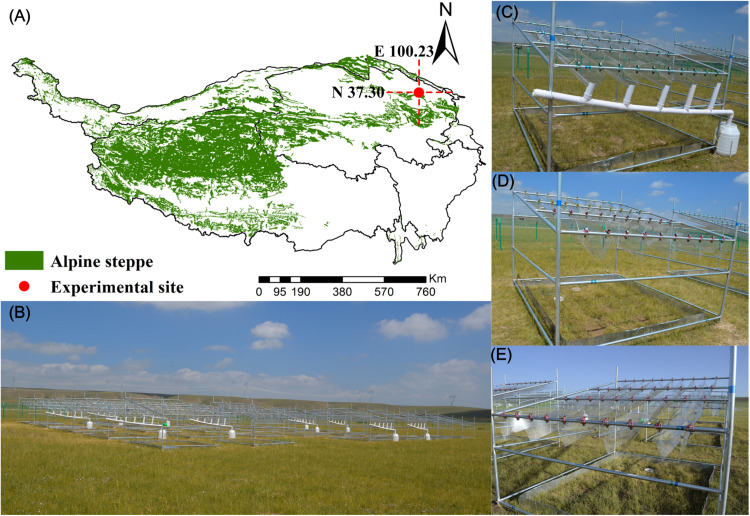
Geographical location and photographs of the experimental site. **(A)** Geographical location; **(B)** overview of the experimental site; **(C)** 50% precipitation reduction treatment; **(D)** 50% precipitation addition treatment; **(E)** nitrogen addition treatment.

Precipitation changes also leave imprints on soil microbial community composition and assemblage (Leff et al., 2015; Zhang et al., 2017; Na et al., 2019). Wang et al. (2018) found that precipitation was a main driver for the distribution pattern and community structure of a soil fungal community at a temperate steppe in Inner Mongolia of China. Additionally, the negative effects of nitrogen deposition on biodiversity may be changed by the effects of elevated precipitation (Zhang et al., 2018). Precipitation has been occurring with an increasing trend gradually on the QTP (Yang, 2018), which might alleviate environmental drought pressure and then might benefit soil fungal diversity and community composition. However, the responses of soil fungal community composition, biodiversity, and assemblage process to the individual effect of precipitation change and interactions with nitrogen deposition are still poorly understood on the QTP. An alpine steppe is one of the main vegetation ecosystems on the QTP (Miehe et al., 2011; Figure 1A) and is more sensitive to climate change than other ecosystems (Zheng and Zhao, 2017). Therefore, alpine steppes are predicted to have stronger responses than other ecosystems to atmospheric nitrogen deposition and precipitation changes. In this study, nitrogen deposition and precipitation amounts were manipulated at an alpine steppe. High-throughput sequencing technology, beta diversity null modeling analysis (Powell et al., 2015), and co-occurrence network analysis (Barberán et al., 2012) were employed to investigate soil fungal community composition, diversity, and community assemblage process in response to nitrogen deposition and precipitation changes. The study examined the following questions: (1) whether soil fungal community composition and diversity were altered by nitrogen deposition, precipitation changes, and their interaction; (2) which environmental factors are significantly related with soil fungal community composition; (3) which ecological process, stochastic or deterministic process, played a dominant role in controlling soil fungal community assemblage and whether this dominant role was altered by nitrogen addition and precipitation changes. Through the examination of these questions, the study aimed to predict how the soil fungal community composition, diversity, and community assemblage processes will change under nitrogen deposition and precipitation changes in the future at an alpine steppe and to provide a theoretical basis for anthropogenic nitrogen source input and precipitation changes in alpine steppe management.

## Materials and Methods

### Study Site and Experimental Design

The field experiment was performed in Gangcha County (37°18′N, 100°15′E), which is located in Qinghai Province in the northeast of the QTP, China (Figure 1A). The altitude, mean annual temperature, and mean annual precipitation were 3,286 m, 0.08°C, and 387 mm, respectively. Alpine steppes are the dominant vegetation and comprised *Stipa purpurea* Grisebach, *Poa crymophila* Keng, and *Leymus secalinus* (Georgi) Tzvelev. The soil was classified as calcium soil order, semidry calcium soil suborder, and chestnut soil (Li et al., 2019).

The field experiment was established in 2013 by manipulating nitrogen deposition and precipitation to simulate atmospheric nitrogen deposition and precipitation changes due to climate change in the alpine steppe (Figure 1B). The experiment included six treatments, namely, NP− (ambient N with 50% precipitation reduction), NP (ambient N with ambient precipitation), NP + (ambient N with 50% precipitation increment), N + P− (N addition with 50% precipitation reduction), N + P (N addition with ambient precipitation), and N + P + (N addition with 50% precipitation increment). Five replicates for each treatment were established; thus, a total of 30 plots of 3.3m × 2.7 m in area were randomly separated in a 5 × 6 block design and divided by 2 m-wide buffer zones. V-shaped, sunlight-pervious, concave polyvinyl chloride (PVC) boards without slots, which were installed above treatments of 50% reduction in precipitation at a 15° angle, were used to collect precipitation (Figure 1C). This precipitation was then transferred (sprinkling evenly) to the treatments of 50% increase in precipitation. For precipitation increments and ambient precipitation treatments, V-shaped, sunlight-pervious, concave PVC boards with slots were established above these plots (Ma et al., 2017) (Figures 1D,E). NH_4_NO_3_ was fertilized in the plots to simulate atmospheric nitrogen deposition (10 g N m^–2^ year^–1^). Although nitrogen deposition will go up to 40 g N m^–2^ year^–1^ by 2050 (Zong et al., 2016), 8 g m^–2^ year^–1^ has led to N saturation in the soil in our study area concurrently (Peng and Yang, 2016). NH_4_NO_3_ was dissolved in 1 L of distilled water and sprinkled evenly twice into the plots of nitrogen addition during the peak growing season (midmonth of June and July). Identical amounts of distilled water were sprayed uniformly on the ambient nitrogen addition plots (Li et al., 2019).

### Plant Community Investigation and Soil Sampling

Species richness under the different treatments was investigated in August (peak of biomass) using a 1 m^2^ quadrat and three replicates for each plot. The total species (non-repeat count) occurring in all three quadrats was used to represent the species richness for each plot. Numbers of each individual species were also recorded. Average relative abundance based on the three quadrats was calculated to represent abundance of each species. For soil sampling, five topsoil cores (0–10 cm) were randomly collected from each plot in August 2019 using a soil auger (diameter: 3.5 cm, depth: 15 cm) with sterilization. The five cores were mixed thoroughly using a 2.0 mm sieve to form a mixing sample without roots and debris, which was transported (at 4°C) to the laboratory within 24 h. Each mixing sample was divided into two parts; one was stored at −80°C for DNA extraction, and the other part was air-dried for analysis of soil physicochemical properties.

### Soil Physicochemical Analyses

Soil samples were dried at 105°C for 48 h to a constant weight to assess soil moisture. Soil pH was determined using a pH meter in a liquid supernatant with a soil-to-water ratio of 1:2.5. Soil organic carbon (SOC) contents were analyzed using a dichromate oxidation-titration method. Soil mineralizable nitrogen (N) and soil available phosphorus (P) and potassium (K) contents were examined using the diffusion by alkali dissociation method, the ammonium molybdate method after extraction with NaHCO_3_ solution, and the flame atomic absorption spectrometric method, respectively (Bao, 2005). Soil mineralizable nitrogen (N), available phosphorus (P), and available potassium (K) represent plant available N, P, and K contents in soil, respectively.

### DNA Extraction, Sequencing, and Bioinformatics Analysis

An E.Z.N.A. Soil DNA Kit (Omega Bio-tek, Inc., Norcross, GA, United States) was used to extract soil genomic DNA from exactly 0.3 g of fresh soil according to the manufacturer’s instructions; three replicates for each soil sample were established. The concentration and purity of the obtained DNA sample were determined using a NanoDrop 2000 spectrophotometer (Thermo Scientific, Wilmington, DE, United States). Primers ITS1F (5′-ACTTGGTCATTTAGAGGAAGTAA-3′) and ITS2 (5′-BGCTGCGTTCTTCATCGATGC-3′) were used to amplify fungal internal transcribed spacer-1 (ITS1) sequences (Adams et al., 2013). Details of the processes and reaction conditions for ITS1 amplification have been described in previous studies (Adams et al., 2013). Sequencing of DNA samples was conducted on an Illumina MiSeq PE300 instrument (Illumina, San Diego, United States) provided by Shanghai Majorbio Bio-Pharm Technology Co., Ltd.

Paired-ended sequence reads generated from the Illumina MiSeq PE300 were filtered by fastp (version 0.20.0; Chen et al., 2018) and merged using FLASH software (version 1.2.11; Magoč and Salzberg, 2011) with the following criteria: (i) 300 bp reads were truncated at any site receiving an average quality score of <20 over a 50 bp sliding window, and truncated reads shorter than 50 bp were discarded; reads containing ambiguous characters were also discarded, and samples were distinguished according to the barcode and primers; (ii) only overlapping sequences longer than 10 bp were assembled according to their overlapped sequence. The maximum mismatch ratio of the overlap region is 0.2. Reads that could not be assembled were discarded; (iii) the sequence direction was adjusted, with exact barcode matching and two-nucleotide mismatch in primer matching. A total of 1,862,202 (99.31%) sequences were obtained for the soil fungal community. Subsequently, operational taxonomic units (OTUs) were generated by clustering the sequences with 97% similarity using UPARSE (version 7.1; Edgar, 2013). The BLAST tool and UNITE database (version 8.0; Koljalg et al., 2013) was used to filter out potentially non-fungal taxa, and then the RDP Classifier version 2.2 (Wang et al., 2007) was used to annotate the most representative sequence of each OTU at a confidence threshold of 0.7. Finally, 3,423 OTUs, which were classified as 13 phyla, 45 classes, 101 orders, 220 families, and 416 genera, were detected across all samples. FUNGuild was used to match fungal functional profiles (Nguyen et al., 2016). All representative sequences in the study were deposited in the GenBank database with accession number PRJNA638547.

### Diversity and Phylogenetic Structure of Soil Fungal Community

A rarefaction curve was used to evaluate sequencing depth (shown in Supplementary Figure S1). The OTU richness, Chao 1 index, and Shannon–Wiener index were used to assess alpha diversity of the soil fungal community. The Shannon–Wiener index evaluates species richness and evenness and is more sensitive to dominant species, whereas Chao 1 index is more sensitive to rare species. The R environment with the functions *chao1*() in the “fossil” package (Vavrek, 2011) and *rowSums*() and *diversity*() in the “vegan” package (Oksanen et al., 2019) was used to calculate the above diversity indices.

Abundance-based β-diversity null modeling analysis with community Bray–Curtis dissimilarity was used to detect soil fungal community assembly processes by evaluating the difference between “real” community and null modeling (neutral process) (Beck et al., 2015; Powell et al., 2015; Powell and Bennett, 2016). This difference was represented by effect size, which was calculated as follows (Mori et al., 2015):

Effect size = [β-diversity_*obs*_ − mean(β-diversity_*null*_)]/β-diversity_*sd*_.

The β-diversity_*obs*_ represented the observed value of a “real” community, and the mean (β-diversity_*null*_) represented the mean of null β-diversity distribution. The β-diversity_*sd*_ represented the standard deviation of null β-diversity distribution. To get the null β-diversity distribution, each OTU in the community was drawn 999 times with the same probability from the OTU pool, which was represented by all of the OTUs occurring in the study. If effect size (relative to neutral prediction) was significantly greater (divergence) or lesser (convergence) than zero, it indicates that the deterministic processes (i.e., interspecific competition or environmental filtering) play leading roles in community assembly. If there was no significant difference between effect size and zero (random), it indicates that neutral factors (for example, dispersal limitations and homogenizing dispersal) control community assembly (Beck et al., 2015; Powell et al., 2015; Powell and Bennett, 2016). The above calculation processes were performed in R (R Core Team, 2015).

### Statistical Analysis

Two-way analysis of variance (ANOVA) was used to determine the effects of nitrogen addition, precipitation alteration, interactions of nitrogen addition and precipitation alteration, and blocking variable on soil environmental factors, plant species richness, and relative abundance of fungal taxon. Significant differences of soil environmental factors, plant species richness, and relative abundance of fungal taxon (Supplementary Table S1) between treatments were examined using Tukey’s test at *P* < 0.05. To estimate the effects of treatments on soil fungal community composition, non-metric multidimensional scaling (NMDS) analysis was used to present visually the differences in soil fungal community composition. Additionally, permutational multivariate ANOVA (PERMANOVA) was conducted based on the Bray–Curtis distance matrix of community relative abundance using the *adonis*() function in the “vegan” package (Oksanen et al., 2019) to determine the effects of nitrogen addition, precipitation changes, nitrogen addition × precipitation changes, and random effect of block (blocking variable) on differences in soil fungal community composition. Spearman’s correlation analysis using the *cor.test*() function was calculated to determine relationships between fungal community composition and environmental factors, and the response variable was the first axes of the NMDS.

A co-occurrence network was used also to explore the co-occurrence patterns in the study (Barberán et al., 2012). Before analysis, low-abundance OTUs (relative abundance < 0.005%) were removed. The ***corr.test***() function in the “psych” package (Revelle, 2018) was used to calculate Spearman’s correlation based on the relative abundance table of soil fungal community, and only Spearman’s correlation coefficient > 0.6 or < −0.6 and ***P*** < 0.05 were included in the network (de Vries et al., 2018; Jiao et al., 2020). Gephi (version 0.9.2)^1^ was used to visualize the co-occurrence network.

## Results

### Soil Environmental Factors and Plant Community Characteristics

Significant differences in soil moisture, soil pH, SOC, plant available N, P in soil, and plant species richness, but not in plant available K content in soil, were detected across the treatments (Table 1). Specifically, soil moistures were significantly higher in the precipitation increment treatments than in the precipitation reduction treatments, but there were no significant differences with NP (control) treatment. In comparison with NP (control) treatment, the nitrogen addition with precipitation reduction (N + P−) treatment increased soil mineralizable N but reduced soil pH and plant species richness. SOC concentration was increased by nitrogen addition and was significantly higher than ambient nitrogen treatments. SOC was also the only environmental factor affected by the interaction of nitrogen addition and precipitation change. Soil available P content declined significantly under the nitrogen addition and precipitation increment (N + P +) treatment, but no significant differences were found with the NP (control) treatment (Table 1). Significant differences in plant community structure were also detected under different treatments (Supplementary Figure S2).

**TABLE 1:** Soil environmental factors and plant species richness under nitrogen addition and precipitation changes.

**Treatment**	**Soil moisture (%)**	**Soil pH**	**Soil organic C (g kg^–1^)**	**Soil mineralizable N (mg kg^–1^)**	**Soil available P (mg kg^–1^)**	**Soil available K (mg kg^–1^)**	**Plant species richness**
NP −	13.37 ± 1.08c	7.98 ± 0.06b	17.46 ± 0.72b	265.00 ± 12.38ab	5.01 ± 0.21a	352.55 ± 43.83a	7.80 ± 0.66b
NP (Control)	17.07 ± 0.83ab	8.11 ± 0.08ab	16.85 ± 0.99b	265.20 ± 16.13ab	3.66 ± 0.68bc	316.80 ± 59.48a	11.40 ± 1.08a
NP +	19.33 ± 1.22a	8.17 ± 0.03a	19.13 ± 1.05ab	241.60 ± 10.22b	3.60 ± 0.27bc	311.59 ± 34.52a	13.60 ± 0.93a
N + P−	14.93 ± 0.39bc	7.75 ± 0.05c	20.18 ± 0.85a	286.80 ± 3.60a	4.82 ± 0.35ab	325.59 ± 29.85a	5.00 ± 0.45c
N + P	16.77 ± 1.30ab	7.99 ± 0.06b	21.07 ± 0.49a	238.20 ± 7.63b	4.35 ± 0.26ab	355.64 ± 50.86a	7.00 ± 0.71bc
N + P +	19.18 ± 0.70a	8.11 ± 0.03ab	18.86 ± 0.45ab	247.40 ± 16.55b	3.08 ± 0.40c	369.62 ± 15.31a	6.60 ± 1.08bc
Significance of							
N	0.718	**0.035**	**0.032**	0.959	0.981	0.461	**0.000**
P	**0.002**	**0.005**	0.981	**0.035**	**0.024**	0.993	**0.004**
N × P	0.661	0.266	**0.035**	0.099	0.355	0.702	0.061
Block	0.931	0.677	0.931	0.430	0.615	0.999	0.334

*Data are mean ± SE (n = 5). Lowercase letters indicate significant differences (P < 0.05). Two-way analysis of variance was used to determine the effect of nitrogen addition (N), precipitation change (P), their interactions (N × P) on observations, and effects of block (Block). The significant differences of these variables between treatments were examined using Tukey’s test at P < 0.05, and significant effects (P < 0.05) are highlighted in bold.*

### Soil Fungi Abundance, Community Composition, and Diversity

**FIGURE 2:**
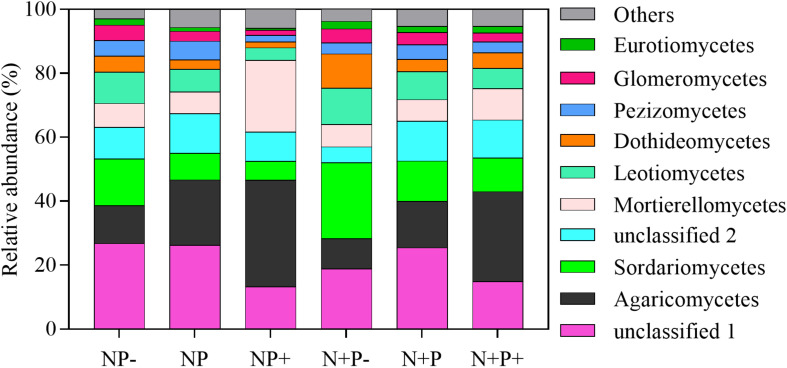
Average relative abundances (%) of the top 10 fungal groups at the class level found at each treatment. Others present the sum of other classes of fungi outside the top 10 in this study. N (ambient N), N + (N addition), P (ambient precipitation), P + (50% added precipitation), and P− (50% reduction in precipitation).

**FIGURE 3:**
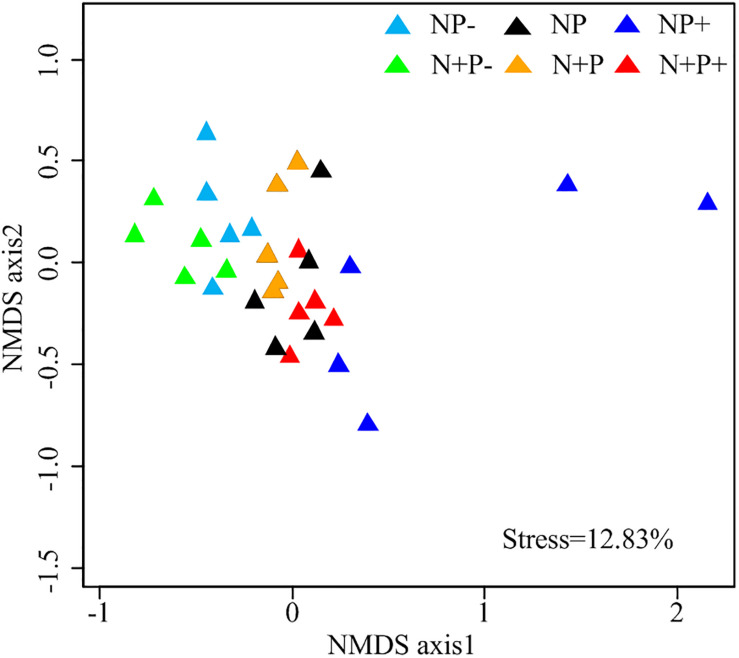
Non-metric multidimensional scaling (NMDS) ordinations based on Bray–Curtis distance dissimilarity matrix of soil fungal Operational taxonomic unit (OTU) taxon composition.

Relative abundances of soil fungi at the class level were significantly different across all the treatments (Figure 2 and Supplementary Table S1). Among groups with a relative abundance > 10.0%, Agaricomycetes and Mortierellomycetes increased significantly under the precipitation increment treatments. In contrast, the abundance of Sordariomycetes declined under the precipitation increment treatments but increased significantly under nitrogen addition with precipitation reduction treatment (Figure 2 and Supplementary Table S1). Significant differences in fungal community composition were also detected under nitrogen addition, precipitation change, and interactive effects of nitrogen addition and precipitation change (Figure 3 and Table 3), in which precipitation change has the highest significant effect on community composition (Table 3).

**FIGURE 4:**
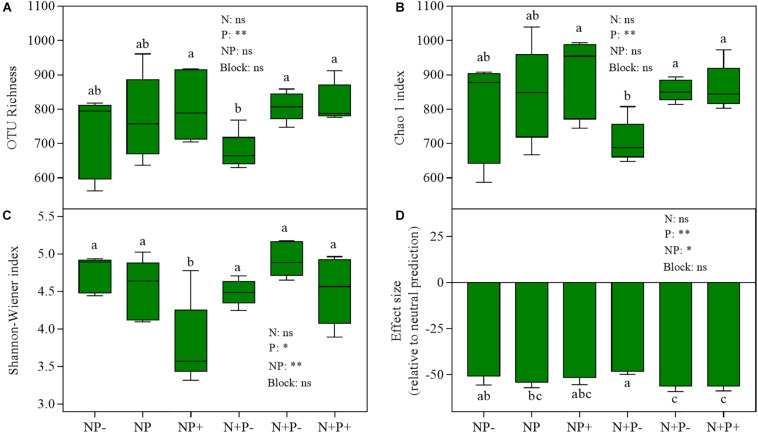
Diversity and effect size relative to neutral prediction based on null model analysis of Bray–Curtis dissimilarity of soil fungal community across treatments. **(A)** OTU richness; **(B)** Chao 1 index; **(C)** Shannon–Wiener index; **(D)** effect size (relative to neutral prediction). Lowercase letters indicate significant differences (*P* < 0.05). Student’s *t*-test was used to determine the difference between effect size of community assembly and zero, and significant differences of these diversity indicators between treatments were examined using Tukey’s test at *P* < 0.05.

Diversity analysis revealed that OTU richness and the Chao 1 index were lower under nitrogen addition combined with precipitation reduction (N + P−) than under control (NP) and other treatments (Figures 4A,B), and the Shannon–Wiener index declined as a result of ambient nitrogen with precipitation increment treatment (Figure 4C). Precipitation change has a significant effect on all three indices, while the interaction of nitrogen addition and precipitation change has a significant effect only on the Shannon–Wiener index (Supplementary Table S3).

### Relationships Between Soil Fungal Community Composition and Soil Environmental Factors and Plant Species Richness

Spearman’s correlation analysis revealed that fungal taxon compositions were related positively and significantly to soil moisture content, soil pH, and plant species richness but were negatively and significantly related to plant available N and P content in soil. There were no significant relationships detected with SOC and plant available K content in soil (Table 2).

**TABLE 2:** Spearman’s correlation analysis between soil fungal community composition and soil environmental factors and plant species richness, and the response variable was the first axes of the non-metric multidimensional scaling (NMDS).

**Explanatory factor**	**Operational taxonomic unit (OTU) community composition**	
	
	***r***	***P***
Soil moisture	**0.5283**	**0.0027**
Soil pH	**0.4995**	**0.0049**
Soil organic C	−0.1275	0.5018
Soil available N	**−0.4425**	**0.0143**
Soil available P	**−0.4818**	**0.0070**
Soil available K	−0.2289	0.2238
Plant species richness	**0.5366**	**0.0022**

*Significant correlations and P-values are in bold type.*

### Community Assembly of the Soil Fungal Community

**FIGURE 5:**
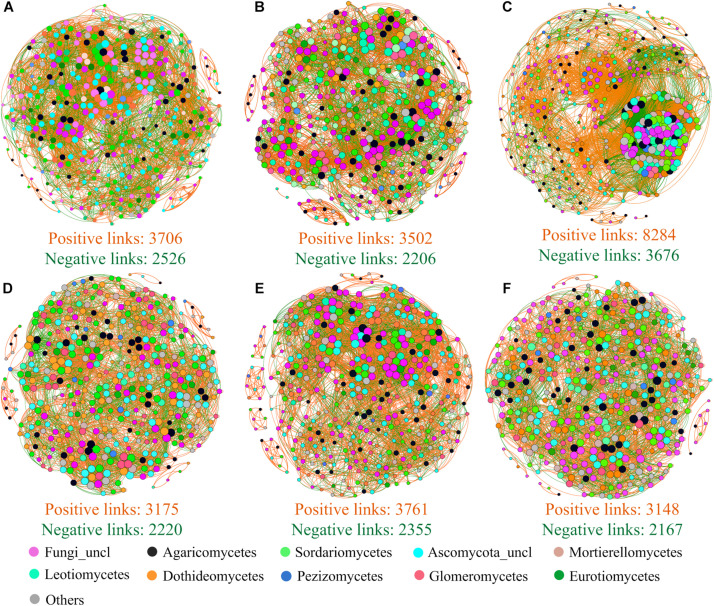
Co-occurrence patterns of soil fungal taxon at OTU level for NP− treatment **(A)**, NP treatment **(B)**, NP + treatment **(C)**, N + P− treatment **(D)**, N + P **(E)**, and N + P + treatment **(F)**. The size of nodes was proportional to the link numbers of each node. Nodes in different colors are representative of the top 10 classes and other classes. Edges are shown with orange lines (positive interaction) and green lines (negative interaction). In each subfigure, only the top 400 OTUs and edges with Spearman’s ρ > 0.60 or < −0.6 and *P* < 0.05 are shown.

Beta diversity null modeling analysis showed that an effect size lesser than zero across treatments (Figure 4D) indicated that deterministic ecological processes (i.e., environmental filtering) were more important than neutral processes in soil fungal community assembly. Compared to that under NP (control) treatment, the importance of environmental filtering was decreased under N + P− treatment (Figure 4D). The co-occurrence network analysis of fungi revealed that strong positive interactions among fungi OTUs were detected under all treatments (Figure 5), in which this positive interaction under NP + treatment was overwhelmingly stronger than all other treatments including NP (control) treatment. The mean of clustering coefficients was up to 0.639, which was higher in NP + treatment than in NP (control) treatment and was lesser in NP treatment than in other treatments (Supplementary Table S4). Other network parameters are shown in Supplementary Table S4.

## Discussion

### Effects of Nitrogen Addition and Precipitation Changes on Relative Abundance and Diversity of Soil Fungi

Several studies have shown that both nitrogen addition and precipitation changes have significant impacts on the relative abundance and diversity of soil fungi (Leff et al., 2015; Chen et al., 2017; Zhang et al., 2018). Here, experimental results also indicated that the relative abundances of soil fungal taxa were altered significantly under nitrogen addition and precipitation alteration (Figure 2 and Supplementary Table S1), suggesting that shifts in both nitrogen and precipitation may influence the ecological functions of soil fungal taxa in the alpine steppe ecosystem. Soil fungi play important roles in the interactions between plants and soil (Semchenko et al., 2018), especially in nutrient acquisition for plants (Bowles et al., 2018). Nitrogen addition and precipitation changes would induce changes in soil availability of nitrogen, soil moisture content, and soil pH (Serna-Chavez et al., 2013; Lu et al., 2014). These changes in soil environmental factors might decrease the importance of some fungal groups involved in nutrient absorption for plants (Leff et al., 2015) and result in alteration of the initial carbon allocation strategy of plants to fungi (Wei et al., 2013). The changes would motivate significant shifts in the relative abundance of soil fungi. In addition, significant changes in plant communities may be another explanation for shifts in the relative abundance of soil fungi (Qin et al., 2020), since plant community changes would trigger shifts in soil fungal trophic groups (e.g., pathogenic and saprophytic fungi) (Makiola et al., 2019).

**FIGURE 6:**
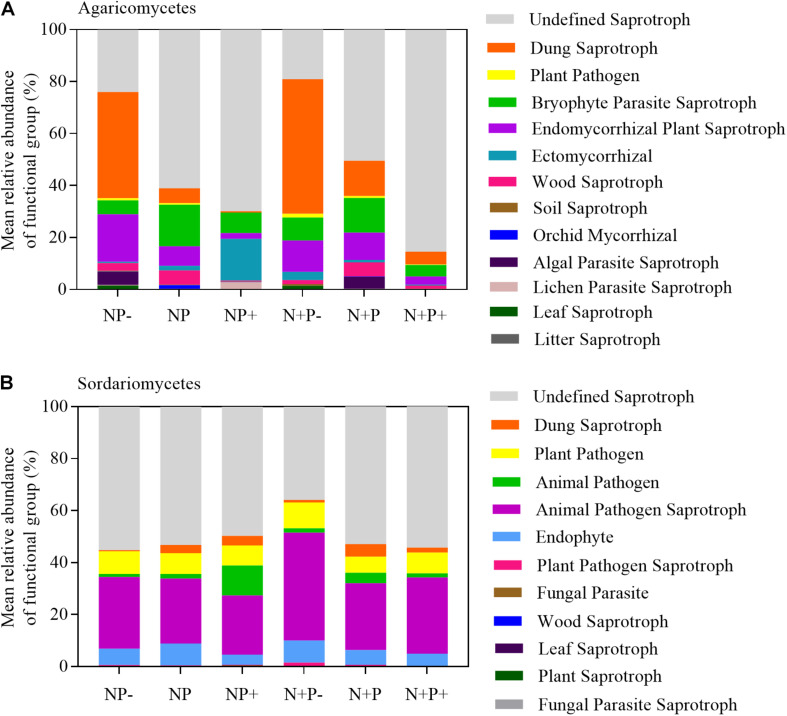
Compositions of fungal functional group (guild) inferred by FUNGuild, Agaricomycetes **(A)** and Sordariomycetes **(B)**.

The relative abundance analysis also revealed a dynamic shift in Agaricomycetes and Sordariomycetes, with one falling and the other rising with the increasing gradient in precipitation (Figure 2). Specifically, the precipitation increment increased the relative abundance of Agaricomycetes but caused a decrease in that of Sordariomycetes. Furthermore, the relative abundance of Sordariomycetes increased in drought conditions. These observations suggested that the two taxa may be related to soil moisture. Results of function prediction showed that Agaricomycetes was closely related to dung saprotroph and plant saprotroph, whereas Sordariomycetes was closely related to soil animal pathogen (Figure 6). A previous study showed that soil animal abundance increased with increasing soil moisture (Sylvain et al., 2014), suggesting that the precipitation reduction treatment might enhance the likelihood of soil animals being infected by pathogens in alpine steppes (Delgado-Baquerizo et al., 2020). However, a study conducted in a subtropical forest reported that the abundance of Agaricomycetes increased in drought conditions (He et al., 2017), which was not consistent with our results. Agaricomycetes contained more undefined function groups (Figure 6A). Therefore, the responses of Agaricomycetes to precipitation may vary in different habitats.

Negative effects of nitrogen deposition on biodiversity, including microbial diversity, have been routinely observed (McClean et al., 2011; Zeng et al., 2016). In this study, nitrogen addition only slightly decreased the diversity of the soil fungal community (Figures 4A–C). However, significant negative effects of nitrogen addition combined with precipitation reduction on OTU richness and Chao 1 index were detected (Figures 4A,B), suggesting that drought reinforces the negative effect of nitrogen addition on soil microbial diversity in the alpine steppe ecosystem. A previous study showed that nitrogen addition combined with elevated precipitation is beneficial for maintaining soil fungal diversity in the *Stipa baicalensis* temperate steppe (Zhang et al., 2018). Based on these observations, we speculated that, in the alpine steppe, drought had a stronger negative effect on microbial diversity than that of nitrogen addition. Moreover, only the Shannon–Wiener index decreased in precipitation addition, which may be related to the increase in the abundance of dominant soil fungal taxa under precipitation increment treatments (Figure 2).

### Relationships Between Soil Fungal Community Composition and Environmental Factors Under Nitrogen Addition and Precipitation Alteration

Soil fungal community composition differed under nitrogen addition and precipitation change (Figure 3 and Table 3), which evidenced the responses of soil microbial composition to nitrogen deposition and precipitation change in the alpine steppe and may be related with the remission of nitrogen and precipitation limitation on the QTP (Zheng and Zhao, 2017). Nitrogen deposition and precipitation change could induce changes in availability of soil nitrogen, moisture, and other soil variables (Serna-Chavez et al., 2013; Xu et al., 2015) and subsequently affected soil fungal community composition (Zhang Y. et al., 2016). This result also further highlighted the significance of this study.

**TABLE 3:** Results of permutational multivariate analysis of variance (PERMANOVA) for fungal community composition, and significant effects (*P* < 0.05) are highlighted in bold.

	**Fungal community composition**		
	***F* model**	***R*^2^**	***P*-value**
N	1.7505	0.0487	**0.016**
P	3.1996	0.1781	**0.001**
N × P	1.3753	0.0765	**0.045**
Block	1.2601	0.1403	0.062

The soil moisture was strongly positively related with soil fungal community composition (Table 2). Precipitation driving the distribution pattern and community structure of a soil fungal community was reported at a temperate steppe (Wang et al., 2018). On the QTP, precipitation change altered the availability of soil moisture at an alpine steppe (Table 1), which may be the main reason for our result. Nitrogen addition would alter the availability of soil nitrogen and induces shifts in plant available P content in soil (Deng et al., 2017; Li et al., 2020) and then changed soil nutrient conditions for soil microbial community. These explained the significant correlation between soil fungal community composition and plant available N and P content in soil (Table 2). Soil pH was an important predictor for soil microbial community composition (Shen et al., 2013; Chen et al., 2017; Zhou et al., 2020) and played primary roles in community assemblage (Dumbrell et al., 2010; Wang et al., 2017; Tripathi et al., 2018; Cho et al., 2019). A significant positive relationship between soil pH and fungal community composition was also revealed (Table 2), which was consistent with previous studies. Change in plant species richness intuitively reflected plant community shift and also significantly related with fungal community composition, which could be explained by the shift in initial carbon allocation strategy of plants to fungi (Wei et al., 2013).

### Mechanism of Soil Fungal Community Assemblage Under Nitrogen Addition and Precipitation Alteration

Beta diversity null modeling analysis has increasingly been employed to access the mechanism of community assemblage (Beck et al., 2015; Powell et al., 2015; Powell and Bennett, 2016). Here, the effect size of null modeling analysis showed that the soil fungal community was convergent across all treatments (Figure 4D), suggesting that deterministic ecological processes, which are largely environmental filtering, play primary and dominant roles in shaping the soil fungal community (Powell and Bennett, 2016). In addition to the N + P− treatment, non-significant differences between others and NP (control) treatment were revealed, suggesting that overall soil fungal community assembly processes may be relatively stable. A previous study has shown that fungal communities are more stable than other microbial groups under climate change (de Vries et al., 2018). The long formation period of alpine steppes may be another reason for a relatively stable process of soil fungal community assemblage under climate change scenarios, since ecosystem development strongly influences belowground biodiversity (Delgado-Baquerizo et al., 2019). The 7-year period of this field experiment was extremely short compared with the long period of alpine steppe formation in the QTP that may have led to a relatively stable process in soil fungal community assemblage. Similarly, a previous study reporting no significant responses of a fungal community to short-term changes in climate conditions (Zhang K. P. et al., 2016) supported our explanation. It should be noted that even though soil fungal communities were convergent across treatments, precipitation change and its interaction with nitrogen addition had significant effects on the community assembly process (Supplementary Table S3), which indicated that soil fungal community assembly processes may tend to neutralize processes under long-term nitrogen deposition and precipitation reduction in the future.

Co-occurrence network analysis is another powerful tool to explore the mechanism of microbial community assemblage (Barberán et al., 2012; Cardinale et al., 2015; Jiao et al., 2020). Here, stronger positive interactions between fungal OTUs were presented (Figure 5), suggesting a closer cooperation among fungal OTUs (Deng et al., 2012) and reflecting, to some extent, that soil fungi might tend to share similar soil niches. Interestingly, precipitation increment (NP +) treatment strongly enhances cluster degree than did control (NP) and other treatments (Figure 5C), which suggested that precipitation increment might trigger a soil ecological process and improve interactions of soil fungal taxon. The mean of clustering coefficients was up to 0.639, which indicated closer relationships between OTUs (Jiao et al., 2020). These speculations might support results that deterministic processes played more important roles in shaping soil fungal community at the alpine steppe on the QTP than neutral ecological processes. Although co-occurrence network analysis was used to infer biotic interactions, we acknowledged shortcomings of the network analysis due to difficulty in accurately detecting community assembly ecological processes (Carr et al., 2019). Therefore, incorporating more experiments or more sophisticated approaches into the research framework will be necessary in the future.

## Conclusion

This study explored the responses of soil fungal community composition, diversity, and community assembly processes to nitrogen addition and precipitation changes at an alpine steppe on the northeastern Qinghai-Tibetan Plateau. Soil fungal community composition and diversity were altered significantly by nitrogen addition and precipitation change, while the convergent processes of fungal community assembly were also altered, even though the dominance of deterministic ecological processes was not changed. The study suggested that soil fungal communities at alpine steppes on the QTP may change a lot under long-term global change scenarios in the future. Future works investigating more comprehensive environmental factors and using more research methods simultaneously are essential to fully understand the construction of fungal communities in nature.

## Data Availability Statement

The datasets presented in this study can be found in online repositories. The names of the repository/repositories and accession number(s) can be found in the article/Supplementary Material.

## Author Contributions

YX, CL, and GZ designed the experiments. YX and GZ jointly wrote the manuscript. YY assisted with the field sampling. YP and YY participated in the discussions. All authors contributed to the article and approved the submitted version.

## Conflict of Interest

The authors declare that the research was conducted in the absence of any commercial or financial relationships that could be construed as a potential conflict of interest.

## Footnotes

1 https://gephi.org/
