# Supplementary material for: Soil Fungal Community Composition, Not Assembly Process, Was Altered by Nitrogen Addition and Precipitation Changes at an Alpine Steppe
Source: Front Microbiol. 2020 Oct 16;11:579072. doi: 10.3389/fmicb.2020.579072 (PMC7597393; doi:10.3389/fmicb.2020.579072)
Supplement: Supplementary file 1 [file Data_Sheet_1.docx]

***Supplementary Material***

1. **Supplementary Figures and Tables**
   1. **Supplementary Figures**

**
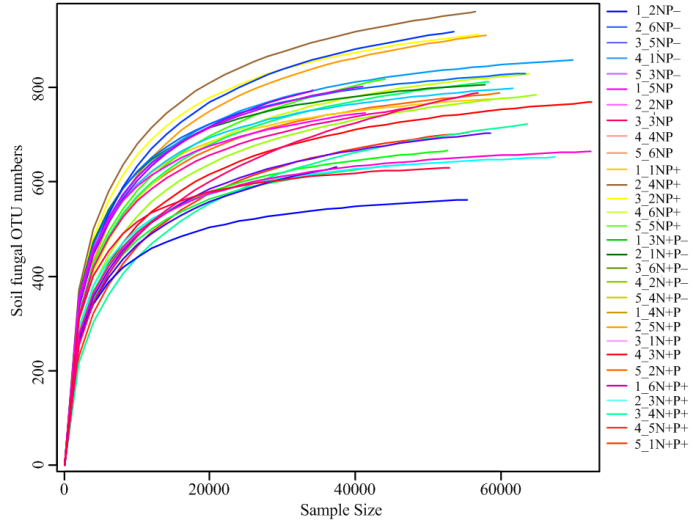
**

**Figure S1** Rarefaction curves for evaluating sequencing depth, and showing sequencing depth is sufficient.


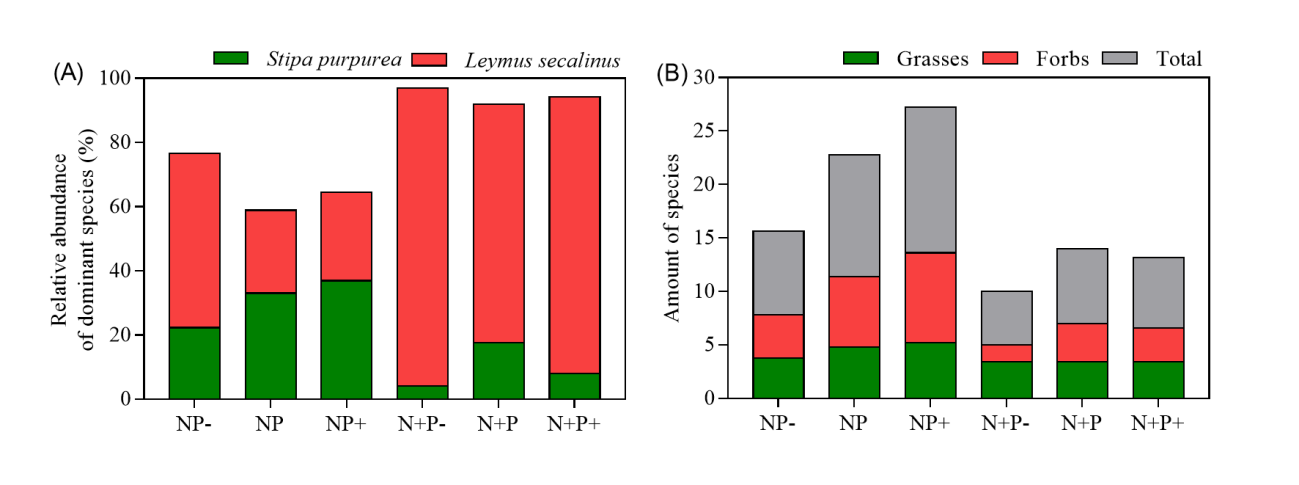


**Figure S2** Relative abundance of dominant species (A) and species richness of different functional groups (B) across treatments at alpine steppe.

- 1. **Supplementary Tables**

**Table S1** Differences of relative abundance (%) of top ten fungal groups at the class level found at each treatment, and the others present the sum of other classes of fungi outside the top ten in the study. The significant differences of these diversity indicators between treatments were examined using Tukey's test at *P* < 0.05.

| Treatments | | Unclassified 1 | Agaricomycetes | Sordariomycetes | Unclassified 2 | Mortierellomycetes | Leotiomycetes | Dothideomycetes | Pezizomycetes | Glomeromycetes | Eurotiomycetes | Others |
| --- | --- | --- | --- | --- | --- | --- | --- | --- | --- | --- | --- | --- |
| NP− | 26.68±  4.26a | 11.92±  2.32c | 14.59±  1.71b | 9.88±  2.39ab | 7.48±  1.32b | 9.85±  1.92ab | 4.96±  1.11ab | 4.90±  1.50a | 4.75±  0.40a | 2.05±  0.29a | 2.93±  0.58a |  |
| NP | 26.17±  7.24a | 20.45±  4.88bc | 8.33±  0.89bc | 12.43±  1.39a | 6.74±  1.77b | 7.13±  0.70bc | 2.96±  0.41b | 5.87±  2.42a | 3.05±  0.73bc | 1.21±  0.19b | 5.67±  1.21a |  |
| NP+ | 13.24±  5.25a | 33.29±  7.81a | 5.90±  1.11c | 9.21±  2.50ab | 22.38±  10.14a | 3.89±  0.41c | 1.91±  0.18b | 2.02±  0.51a | 1.59±  0.45c | 0.61±  0.08b | 5.96±  2.61a |  |
| N+P− | 18.83±  5.69a | 9.50±  1.28c | 23.72±  4.99a | 4.94±  0.20b | 7.00±  1.01b | 11.39±  1.81a | 10.69±  5.11a | 3.50±  1.52a | 4.32±  0.80ab | 2.32±  0.24a | 3.81±  0.46a |  |
| N+P | 25.40±  4.43a | 14.58±  0.98c | 12.54±  1.47bc | 12.48±  1.61a | 6.74±  0.39b | 8.81±  0.86ab | 3.82±  0.78ab | 4.56±  2.11a | 3.73±  0.27ab | 1.94±  0.21a | 5.39±  0.61a |  |
| N+P+ | 14.78±  2.96a | 28.16±  3.22ab | 10.61±  1.06bc | 11.82±  0.74a | 9.85±  1.30ab | 6.31±  0.70bc | 4.85±  1.05ab | 3.44±  2.17a | 2.83±  0.31bc | 2.01±  0.20a | 5.34±  0.84a |  |
| Mean (%) | 20.85 | 19.65 | 12.62 | 10.13 | 10.03 | 7.90 | 4.87 | 4.05 | 3.38 | 1.69 | 4.85 |  |

**Table S2** Summary of ANOVAs for fungal relative abundance at class level

|  | Unclassified 1 | | Agaricomycetes | | Sordariomycetes | | Unclassified 2 | | Mortierellomycetes | |
| --- | --- | --- | --- | --- | --- | --- | --- | --- | --- | --- |
|  | *F*-value | *P*-value | *F*-value | *P*-value | *F*-value | *P*-value | *F*-value | *P*-value | *F*-value | *P*-value |
| N | 0.2950 | 0.5920 | 1.7790 | 0.194 | 9.0990 | **0.0058** | 0.2570 | 0.6169 | 1.4610 | 0.2382 |
| P | 2.6940 | 0.1130 | 23.7570 | **5.16×10^-5^** | 19.9090 | **0.0002** | 2.8690 | 0.1028 | 4.0800 | **0.0500** |
| N×P | 0.7770 | 0.3860 | 0.1090 | 0.744 | 0.8180 | 0.3744 | 4.2340 | **0.0500** | 1.8800 | 0.1825 |
| Block | 0.0490 | 0.8270 | 0.3370 | 0.567 | 0.0420 | 0.8402 | 0.2640 | 0.6120 | 0.3310 | 0.5704 |
|  | Leotiomycetes | | Dothideomycetes | | Pezizomycetes | | Glomeromycetes | | Eurotiomycetes | |
|  | *F*-value | *P*-value | *F*-value | *P*-value | *F*-value | *P*-value | *F*-value | *P*-value | *F*-value | *P*-value |
| N | 4.6520 | **0.0408** | 3.4660 | 0.0744 | 0.0910 | 0.7650 | 1.4070 | 0.2468 | 22.2780 | 7.70**×10^-^**^5^ |
| P | 26.8030 | 2.36**×10^-^**^5^ | 4.5400 | **0.0431** | 0.7080 | 0.4080 | 20.4500 | **0.0001** | 17.7480 | **0.0003** |
| N×P | 0.1730 | 0.6808 | 0.4450 | 0.5110 | 0.6500 | 0.4280 | 2.6890 | 0.1136 | 7.2970 | **0.0123** |
| Block | 0.6150 | 0.2020 | 3.1500 | 0.0842 | 2.4820 | 0.1280 | 0.7510 | 0.3945 | 0.6320 | 0.4343 |

**Table S3** Summary of ANOVAs for fungal community diversity

|  | OTU Richness | | Chao 1 index | | Shannon-Wiener index | | Effect size | |
| --- | --- | --- | --- | --- | --- | --- | --- | --- |
|  | *F*-value | *P*-value | *F*-value | *P*-value | *F*-value | *P*-value | *F*-value | *P*-value |
| N | 0.0010 | 0.9815 | 0.9600 | 0.3367 | 4.0230 | 0.0558 | 0.8410 | 0.3680 |
| P | 8.8830 | **0.0063** | 8.4860 | **0.0074** | 6.7410 | **0.0156** | 6.5140 | **0.0172** |
| N×P | 0.4990 | 0.4863 | 0.4240 | 0.5208 | 7.3350 | **0.0120** | 4.5900 | **0.0421** |
| Block | 2.1630 | 0.1638 | 1.5350 | 0.2268 | 0.6790 | 0.4178 | 0.0130 | 0.9079 |

**Table S4** Parameter summary of the soil fungi co-occurrence network analysis

|  | NP− | NP | NP+ | N+P− | N+P | N+P+ |
| --- | --- | --- | --- | --- | --- | --- |
| Total link numbers | 6232 | 5708 | 11960 | 5395 | 6116 | 5315 |
| Negative link numbers | 2526 | 2206 | 3676 | 2220 | 2355 | 2167 |
| Positive link numbers | 3706 | 3502 | 8284 | 3175 | 3761 | 3148 |
| Number of vertices | 400 | 400 | 400 | 400 | 400 | 400 |
| Average degree | 31.16 | 28.54 | 59.80 | 26.96 | 30.58 | 26.58 |
| Average path length | 3.18 | 3.24 | 2.87 | 3.27 | 3.14 | 3.26 |
| Diameter | 6 | 6 | 6 | 6 | 6 | 6 |
| Clustering Coefficient | 0.619 | 0.638 | 0.724 | 0.618 | 0.632 | 0.608 |
