# Supplementary figures and images for: Soil Fungal Community Composition, Not Assembly Process, Was Altered by Nitrogen Addition and Precipitation Changes at an Alpine Steppe
Source: Front Microbiol. 2020 Oct 16;11:579072. doi: 10.3389/fmicb.2020.579072 (PMC7597393; doi:10.3389/fmicb.2020.579072)

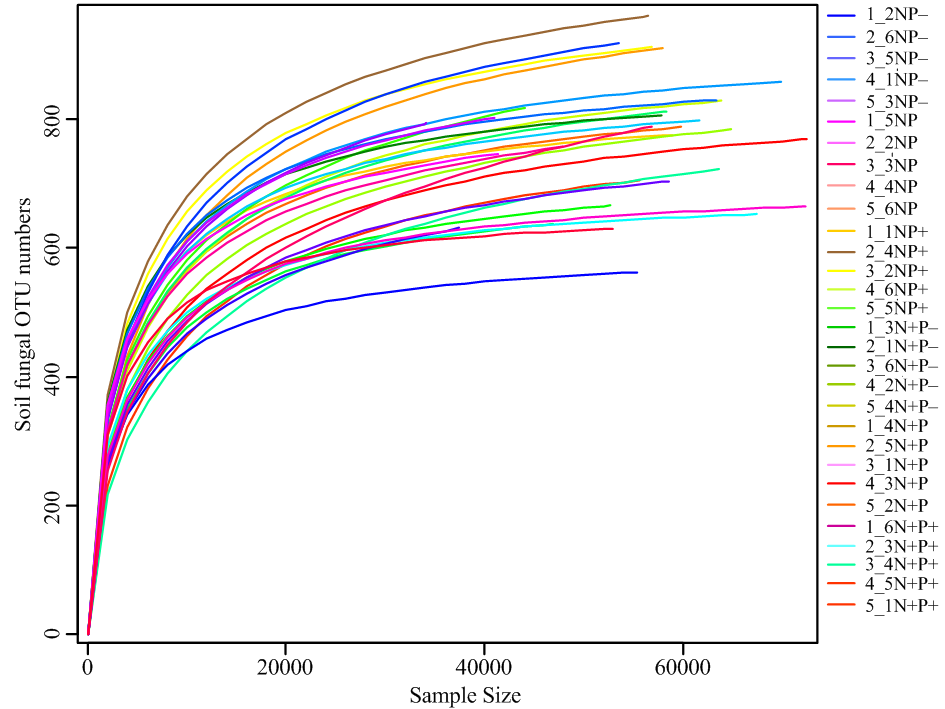

Supplement: Supplementary file 2 [file Image_1.TIF]

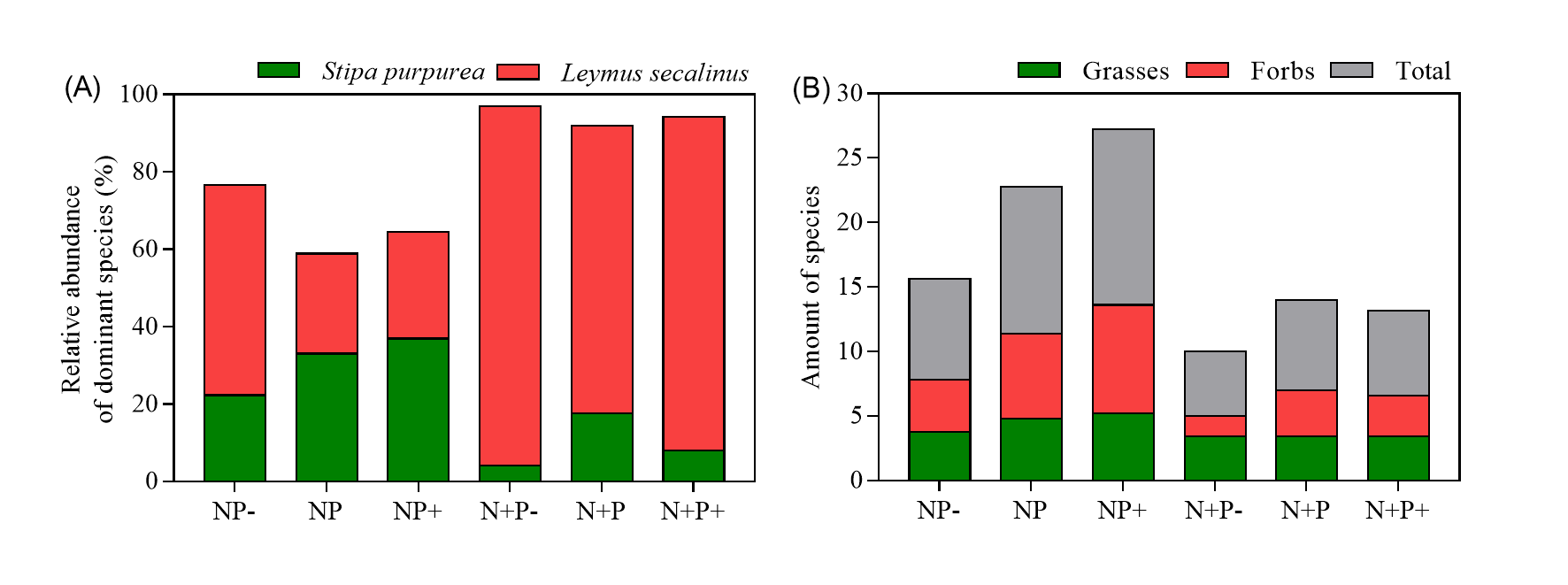

Supplement: Supplementary file 3 [file Image_2.TIF]
